# Effect of Different Chemo-Mechanical Shaping Protocols on the Intratubular Penetration of a Bioceramic Sealer

**DOI:** 10.3390/jcm15031132

**Published:** 2026-02-01

**Authors:** Luigi Generali, Federica Veneri, Carlo Gaeta, Francesco Cavani, Emanuele Ambu, Sara Bertucci, Giuseppina Vallotto, Tommaso Filippini, Eugenio Pedullà

**Affiliations:** 1 Department of Surgery, Medicine, Dentistry and Morphological Sciences with Transplant Surgery, Oncology and Regenerative Medicine Relevance (CHIMOMO), University of Modena and Reggio Emilia, 41124 Modena, Italy; luigi.generali@unimore.it (L.G.); bertuccisara@gmail.com (S.B.); vallottogiusy@gmail.com (G.V.); 2 Department of Medical Biotechnologies, University of Siena, 53100 Siena, Italy; carlo.gaeta@unisi.it (C.G.); emanuele.ambu@unisi.it (E.A.); 3 Department of Biomedical, Metabolic and Neural Sciences, University of Modena and Reggio Emilia, 41124 Modena, Italy; francesco.cavani@unimore.it (F.C.);; 4 Department of General Surgery and Surgical-Medical Specialties, University of Catania, 95131 Catania, Italy; eugeniopedulla@gmail.com

**Keywords:** bioceramic, dentin tubules, irrigation, irrigant activation, sealer penetration

## Abstract

**Background**: This study aimed to evaluate the effect of two shaping systems combined with different irrigant activation methods on the tridimensional distribution of a bioceramic root canal sealer. **Methods**: Sixty single, round, straight root canals from extracted human teeth were randomized into six groups (*n* = 10): A1–A3 shaped with RACE NiTi rotary files; B1–B3 shaped with an adaptive XP-Endo Shaper. NaOCl and EDTA irrigation was performed using passive ultrasonic irrigation (PUI, group 1), conventional endodontic needle irrigation (CENI, group 2), or XP-Endo Finisher mechanical activation (group 3). Canals were obturated using the single-cone cold gutta-percha technique with BioRoot RCS bioceramic sealer. Confocal laser scanning microscopy was used to assess sealer penetration (mean and maximum depths and percentage), and sealer integrity on canal walls at coronal, middle, and apical levels. **Results**: The XP-Endo Shaper combined with the XP-Endo Finisher showed the highest mean sealer penetration depth, while RACE with PUI had the lowest (B3 vs. B2 *p* = 0.02; vs. A1 *p* = 0.05). No significant differences were observed in the maximum penetration depth and percentage of penetration across groups. Sealer integrity was significantly lower in the RACE + XP-Endo Finisher group (*p* < 0.01). Coronal regions consistently showed higher mean and maximum sealer penetration and percentage of penetration compared to apical thirds, with no significant differences in sealer integrity within root regions. **Conclusions**: The combination of the XP-Endo Shaper and XP-Endo Finisher showed a tendency towards superior sealer tridimensional distribution, particularly in the middle and apical thirds. This in vitro study suggests that adaptive shaping instruments combined with mechanical activation enhance sealer distribution, potentially improving treatment success.

## 1. Introduction

The key phases for the long-term success of endodontic therapy include appropriate mechanical shaping, chemical disinfection, and tridimensional obturation of the root canal system [1,2]. Complete disinfection of the root canal system cannot be achieved by mechanical shaping alone, due to its complex anatomy. Therefore, thorough chemical irrigation is required to remove debris and the smear layer, thereby facilitating the penetration of disinfectants and obturation materials into the dentinal tubules [3,4]. The irrigant effectiveness can be increased by a variety of methods. These include activation through manual agitation, heating, negative pressure irrigation, sonic and ultrasonic activation, laser, and activation by rotating NiTi instruments [1,5]. Finally, the placement of a tridimensional obturation is necessary to prevent the root canal recontamination and to achieve the “entombment” of residual bacteria that are inevitably present [6]. In this context, bioceramic sealers have shown some advantageous properties in terms of seal quality, physicochemical properties, antibacterial activity, bioactivity, and biocompatibility [7,8,9]. Both intratubular penetration and peripheral sealer integrity are considered relevant parameters for evaluating obturation quality, as they contribute to sealing ability, antibacterial activity, and micromechanical retention [10,11,12,13].

Recent advances in nickel–titanium instrumentation have introduced adaptive shaping systems designed to improve canal preparation while preserving root anatomy [14,15]. Among these, the XP-Endo Shaper (Reamer with Alternating Cutting Edges, FKG Dentaire, La Chauz-de-Fonds, Switzerland), manufactured from MaxWire alloy, exhibits thermomechanical behavior that allows expansion at body temperature, potentially increasing canal wall contact, compared with conventional rotary systems. Similarly, dedicated instruments such as the XP-Endo Finisher (FKG Dentaire) have been proposed to enhance irrigant activation through mechanical agitation without additional dentin removal.

Conventional rotary systems and passive ultrasonic irrigation remain widely used and are regarded as reference approaches, yet their combined effects with newer adaptive technologies on sealer distribution are not fully understood.

Previous studies have separately investigated the performance of different shaping techniques, irrigants, activation methods, or sealers, mostly regarding smear layer removal [16,17]. However, the effectiveness of different combinations of these on the sealer penetration into dentinal tubules and their tridimensional distribution has not been extensively studied [18,19].

Thus, the aim of the present study was to evaluate the effects of two shaping systems with different kinematics (rotary and adaptive), combined with different irrigant activation methods (passive ultrasonic irrigation (PUI), conventional endodontic needle irrigation (CENI), and mechanical activation), on the tridimensional distribution and intratubular penetration of a bioceramic sealer.

The null hypothesis tested was that the different chemo-mechanical shaping protocols used (i.e., the type of shaping, the type of irrigant activation, and the interaction between these two factors) would yield no significant differences in terms of depth and percentage of sealer penetration into the dentinal tubules and the integrity of the sealer on the canal perimeter.

## 2. Materials and Methods

**Figure 1:**
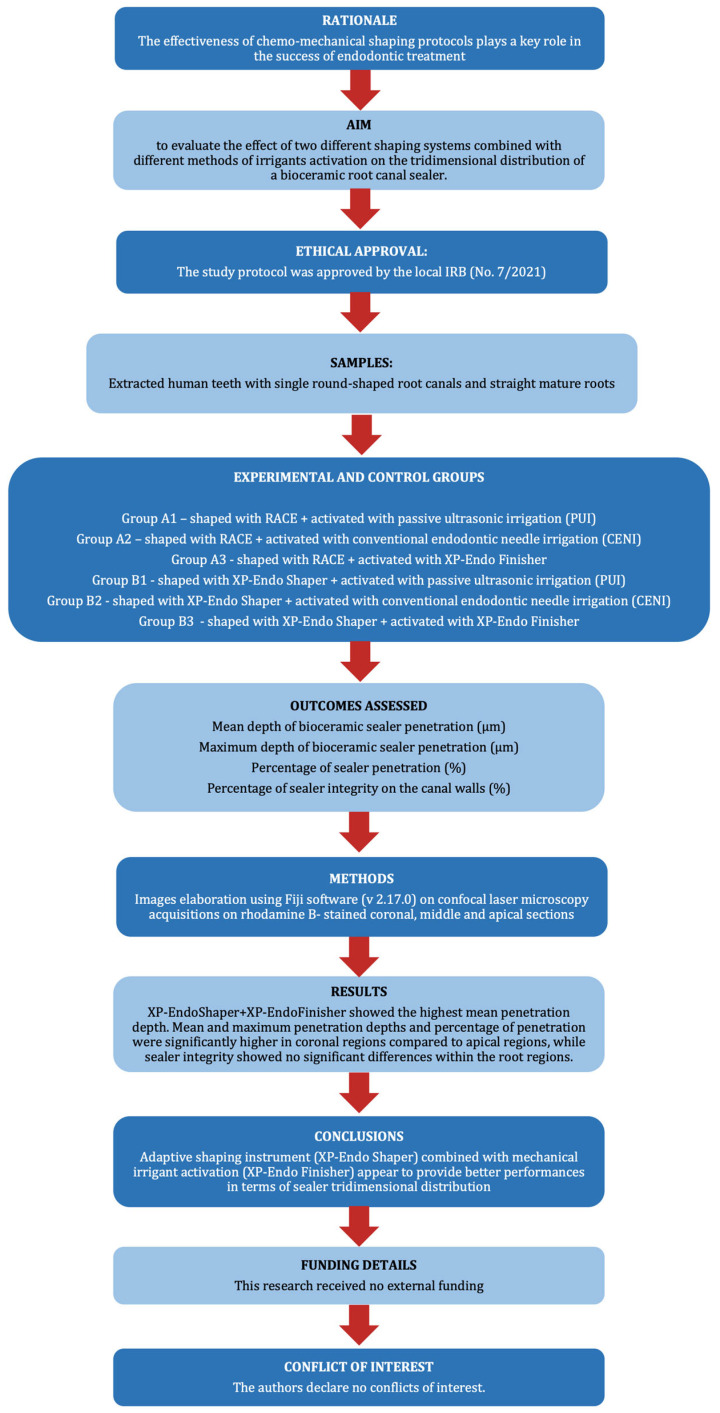
Preferred Reporting Items for Laboratory studies in Endodontology (PRILE) flowchart.

The manuscript of this preclinical study has been written according to the Preferred Reporting Items for Laboratory studies in Endodontology (PRILE) 2021 guidelines. The PRILE flowchart is displayed in Figure 1. The study protocol was approved by the local Institutional Review Board of the Azienda Ospedaliero-Universitaria Senese (No. 7/2021) and performed in accordance with the principles of the Declaration of Helsinki. Informed consent was obtained from all subjects involved in the study: in accordance with the guidelines and regulations of the local ethics committee, the volunteers were informed about the use of their extracted teeth in research, and their oral consent was obtained.

### 2.1. Sample Selection

The sample size was estimated according to previous studies [10,20] and the minimum sample size resulted in 10 teeth for each group for a test power of 0.80 (G*Power 3.1.9.2 software, Heinrich-Heine-Universität Düsseldorf, Düsseldorf, Germany), with α = 0.05. Teeth were obtained from an anonymized biobank of permanent teeth extracted for periodontal reasons; donor age information was not available. The extracted teeth were disinfected in 5.25% sodium hypochlorite (NaOCl)—(Niclor, Ogna, Muggio, Italy) for ten minutes, rinsed with sterile water, then stored in 0.1% thymol solution (Baxter S.p.a, Rome, Italy) at 4 °C until use and for no longer than 15 days. Teeth were inspected under a 10× optical microscope (OPMI Pico, Carl Zeiss Meditec Inc., Jena, Germany) for signs of root fractures, root caries, and resorptions. Schneider’s method was used to measure the degree of canal curvature using digital radiographs acquired in buccolingual and mesio-distal directions. Only straight roots (i.e., with a degree of curvature of less than 10°) [21] were selected. Small-field CBCT scans were obtained to exclude teeth with oval canals or with multiple canals; thus, only mono-radicular teeth with single, round-shaped root canals and straight mature roots were included in the study (*N* = 60).

### 2.2. Working Length and Glide Path

The coronal portions of the sample teeth were removed at the level of the cement-enamel junction (CEJ) using a diamond bur (Komet Dental, Lemgo, Germany) mounted on a high-speed handpiece under continuous water irrigation to obtain standardized roots of 14 ± 1 mm length. The working length (WL) was measured by inserting a 21 mm #10 C-file (Dentsply Maillefer, Ballaigues, Switzerland) until its tip appeared at the apical foramen under microscopic observation at 10×. A mechanical glide path was obtained with NiTi Endowave MGP (J. Morita, Bresso, MI, Italy) n.1 (10/.02), n.2 (15/.02), and n.3 (20/.02) operated at 800 rpm speed and 0.3 N/cm torque. Between each instrument, the root canals were irrigated with 2 mL 5.25% NaOCl using a syringe with a 30-gauge side-vented needle (Irrigation Probe, Kerr Corporation, Orange, CA, USA).

All instruments were used according to the manufacturer’s instructions, mounted on an X Smart plus engine (Dentsply, Maillefer, Ballaigues, Switzerland).

### 2.3. Shaping

Samples were randomized using a computer-generated sequence via a web-based algorithm (www.random.org), which uniquely assigned samples numbered 1–60 to six experimental groups (A1, A2, A3, B1, B2, B3), each comprising 10 teeth, based on the chemo-mechanical shaping procedure. Group A root canals were shaped with RACE NiTi rotary instruments (Reamer with Alternating Cutting Edges, FKG Dentaire, La Chauz-de-Fonds, Switzerland) 20/.04, 25/.04, and 30/.04 used according to the manufacturer’s instructions, i.e., 600 rpm and torque 1.5 N/cm, irrigating the canal with 2 mL of 5.25% NaOCl between each instrument.

Group B root canals were shaped using a NiTi adaptive instrument XP-Endo Shaper (FKG Dentaire, La Chauz-de-Fonds, Switzerland) in the canal filled with NaOCl. The instrument was operated according to the manufacturer’s instructions by inserting it into the canal at 1000 rpm and 1 N/cm torque, with gentle pressure in the apical direction, until the working length was reached with 3 to 5 runs of the instrument. The canals were then rinsed with 2 mL of NaOCl 5.25%, and the instruments were used 15 times at full WL, always immersed in NaOCl.

### 2.4. Final Activation of Irrigants

Root canals from the groups indicated by the number 1 underwent passive ultrasonic irrigation (PUI). Final irrigant activation was performed using the EndoUltra root canal irrigator (InterMed, Alachua, FL, USA). After shaping was completed, the canal was irrigated with 1 mL of 5.25% NaOCl activated by ultrasound for 30 s; then, the canal was irrigated again with 1 mL of NaOCl activated for another 30 s. Then, the canal was irrigated with 1 mL of 17% EDTA activated by ultrasound for 30 s, then irrigated again with 1 mL of 17% EDTA activated for another 30 s. The tip of the ultrasonic insert was used in a continuous apico-coronal motion, ensuring that it did not touch the canal walls. Finally, a rinse with 2 mL of saline was performed to remove chemical residues.

Root canals from the groups indicated by the number 2 underwent conventional endodontic needle irrigation (CENI). After shaping, the canal was irrigated with 1 mL of NaOCl for 30 s through a syringe with a 25-gauge side-vented needle (Irrigation Probe, Kerr Corp, Orange, CA, USA), with the needle inserted deep into the canal but no more apical than 2 mm from the WL. The canal was then irrigated with 1 mL of 17% EDTA for 30 s in the same manner. Both the NaOCl and EDTA irrigations were repeated twice for a total of 60 s each. Finally, the canal was rinsed with 2 mL of saline to remove chemical residues.

Root canals from the groups indicated by the number 3 underwent activation of irrigant using the XP-Endo Finisher (FKG Dentaire, La Chauz-de-Fonds, Switzerland) NiTi instrument. The XP-Endo Finisher was inserted at WL into the canal filled with NaOCl and operated at 1000 rpm and 1 N/cm torque for 60 s. This was followed by irrigation with 17% EDTA solution activated with the same protocol. A final rinse with 2 mL of NaOCl was performed to inactivate the EDTA. Finally, the canal was rinsed with 2 mL of saline to remove chemical residues. All solutions were delivered in the canal with a syringe equipped with a 30-gauge side-vented needle placed 2 mm from the WL. The details of the chemo-mechanical shaping protocols applied are reported in Table 1.

### 2.5. Canal Obturation

Canals were dried by successive insertion of standardized #30 paper cones (Dentsply Maillefer, Ballaigues, Switzerland) at working length, until the tip of the cone removed from the canal and rubbed on a rhodium mirror left no halo.

All specimens were obturated using the single-cone cold gutta-percha technique with BioRoot RCS bioceramic sealer (Septodont, Saint-Maur-des-Fossés, France). Gutta-percha cones with a diameter of #30 and a 4% taper (Inline, BM Dentale sas, Turin, Italy) were used. Rhodamine B was used as a fluorescent tracer to visualize sealer-associated fluorescence under CLSM and to allow standardized comparative assessment among groups. The sealer was prepared according to the manufacturer’s instructions, by mixing the powder component with the liquid and adding Rhodamine B dye (Carlo Erba Reagents, Arese, Italy) at 0.1% by weight to allow later observation of the sealer penetration under a confocal microscope. The dye-labeled sealer was delivered into the canal using a syringe with a disposable Total Fill tip (Busa, Brasseler, Savannah, GA, USA) and distributed throughout the canal by apico-coronal movements of the gutta-percha cone. After obturation was completed, the excess portion of the cone was removed using a heat carrier.

All specimens were then stored in an incubator at 37 °C with 100% humidity for 1 week to allow the sealer to set.

The potential occurrence of perforation, instrument breakage, or specimen damage during procedures was verified by visual inspection under a dental operating microscope (OPMI Pico, Carl Zeiss Meditec Inc., Jena, Germany) at 10× magnification and ultimately recorded as a procedural incident, and the corresponding specimens were discarded. All procedures were performed by the same experienced operator, who was not blinded to the experimental allocation of the samples, due to technical reasons.

### 2.6. Sample Preparation for CLSM Analysis

The specimens were embedded in transparent epoxy resin (Hardrock 554, Remet, Casalecchio di Reno, BO, Italy) under a hood. Three 200-micron-thick transverse sections were obtained from each specimen using a Leica 1600 rotary microtome (Leica SP1600, Leica, Nussloch, Germany) equipped with a diamond blade under continuous water flow. Sections were taken at 2, 5, and 8 mm from the apex, corresponding to the apical, middle, and coronal regions, respectively. These landmarks were selected for standardization reasons, as previously performed in other studies [22,23].

### 2.7. Confocal Laser Microscopy Morphometric Analysis

**Figure 2:**
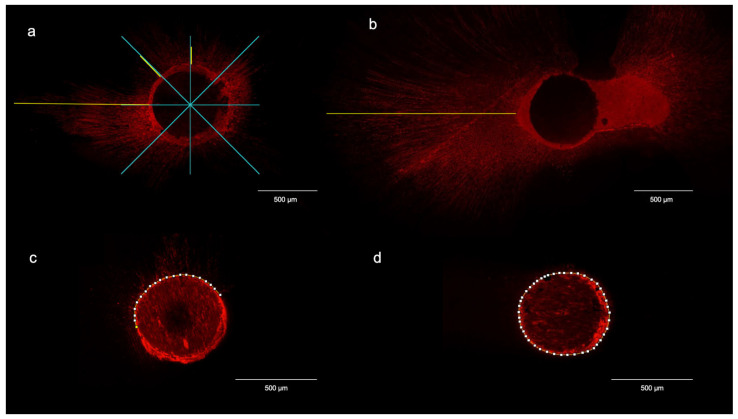
Straight line function of Fiji software used to calculate (**a**) mean depth of sealer penetration and (**b**) maximum sealer penetration into dentinal tubules. Segmented-line function of Fiji software used to calculate (**c**) the percentage of sealer penetration into dentinal tubules and (**d**) the percentage of sealer integrity on the canal perimeter. Image scale is defined by calibrated scale bars rather than nominal magnification to provide a more accurate and absolute spatial reference.

Each section was examined under a confocal laser scanning microscope (CLSM) Leica SP8 AOBS (Leica Microsystems, Mannheim, Germany) equipped with a white light laser using the specific wavelengths for rhodamine B (i.e., excitation at 540 nm and emission at 590 nm). The images were acquired at a 10× magnification and then analyzed using Fiji software (v 2.17.0, National Institutes of Health, Bethesda, MD, USA) to evaluate the penetration of the bioceramic sealer into the dentin tubules (Figure 2).

The penetration depth of the sealer labeled with rhodamine B was measured using the straight-line tool of Fiji software. The mean depth of sealer penetration (µm) was calculated as the average depth at 8 standardized points starting from the inner side of the canal wall for each root region. The point of deepest penetration (µm) was also measured from the canal wall to the point of maximum depth of sealer penetration.

The percentage of sealer penetration was calculated by measuring the rhodamine B–stained surfaces of the canal wall where sealer penetrated inside dentinal tubules (sealer tags) and dividing these values by the circumference of the root canal itself and multiplying the result by 100. Moreover, the percentage of sealer integrity, which indicates the presence of a sealer layer at the perimeter of the canal regardless of penetration into the dentinal tubules, was evaluated on each image by measuring the rhodamine-stained perimeter of the canal wall and dividing this value by the root canal circumference. The segmented-line tool of the Fiji software was used to perform these measurements.

All measurements were performed by the same experienced operator, using standardized protocols, predefined measurement points, and calibrated software tools. The operator was blinded to group allocation during image analysis to minimize bias.

### 2.8. Statistical Analysis

All statistical analyses were performed using Stata 16.1 software (StataCorp Lp, College Station, TX, USA). The Shapiro–Wilk test was used to assess the normal distribution of the data. The comparisons were carried out both among overall groups (e.g., A1 vs. A2 vs. A3, etc.) and among specific root regions. As the data were not normally distributed, comparisons between groups and within each group for sealer penetration at different root regions (expressed as mean, maximum, and percentage) were performed using the nonparametric Kruskal–Wallis, test followed by Dunn’s test with Bonferroni correction for post hoc pairwise multiple comparisons, when appropriate. The interaction between shaping protocol and activation protocol between each other and with the measured parameters was assessed using the rank-transformed two-way ANOVA, followed by the one-way post hoc analysis when appropriate. A *p*-value < 0.05 was considered statistically significant. Although nonparametric tests were used, results are presented graphically as mean ± standard deviation to allow a comparison with the existing literature; corresponding median and interquartile range values are provided in the Supplementary Material.

## 3. Results

### 3.1. Mean Penetration Depth

**Figure 3:**
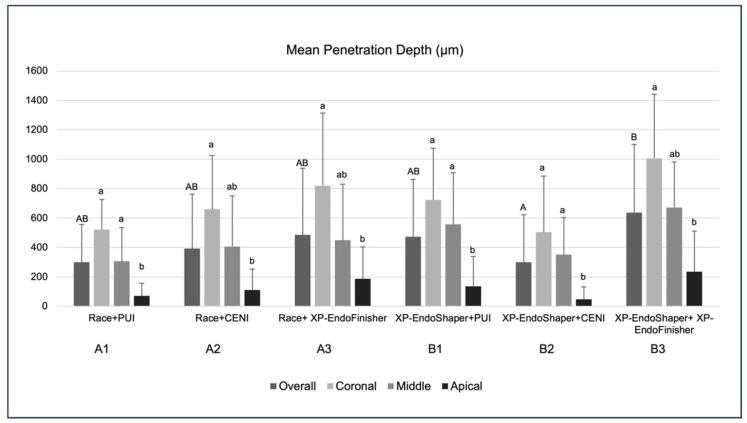
Mean sealer penetration depth (µm) ± standard deviation measured by CLSM in the six experimental groups (*n* = 10 per group) overall and at coronal, middle, and apical root levels. Overall inter-group comparison showed significant differences among groups (Kruskal–Wallis test: groups sharing an uppercase letter are not significantly different; *p* > 0.05). Within-group analysis showed significantly lower penetration percentages in the apical region compared with coronal and/or middle regions (Kruskal–Wallis test: groups sharing a lowercase letter are not significantly different; *p* > 0.05).

No procedural errors or specimen damage occurred; therefore, all samples were included in the analyses, resulting in a total of 60 mono-radicular teeth, with straight and mature roots. Each experimental group included 10 specimens, resulting in a total of 60 round-shaped root canals analyzed. According to the measurements acquired through CLSM morphometric analyses for the mean penetration depth, Group A1 showed the overall lowest value (299.31 ± 257.9 µm), while group B3 showed the highest values (637.84 ± 463.93 µm) of overall mean penetration depth (Figure 3), statistically significant as compared to group B2 (*p* = 0.023) and close to statistically significance compared to group A1 (*p* = 0.053).

No significant differences were detected among the same root regions belonging to different groups.

As for the analysis within groups, the mean penetration depth in apical regions was significantly lower compared to coronal regions in all groups (*p* < 0.05), and also significantly lower compared to the middle regions in groups A1, B1, and B2 (*p* < 0.05); no other significant differences were observed.

According to the multivariate analysis, the type of irrigant activation had a significant effect on the mean depth of sealer penetration. Specifically, XP-EndoFinisher showed the highest values, followed by PUI and CENI. The difference between groups activated with XP-EndoFinisher and CENI was statistically significant (*p* = 0.02). On the other hand, the shaping method had no significant effect on the overall mean depth of sealer penetration (*p* = 0.23).

Inter-group comparison within the same root region showed no statistically significant differences (*p* > 0.05). Intra-group comparisons among root regions demonstrated significantly lower maximum penetration in the apical third compared with coronal and/or middle thirds (groups sharing a lowercase letter are not significantly different; *p* > 0.05).

### 3.2. Maximum Penetration Depth

**Figure 4:**
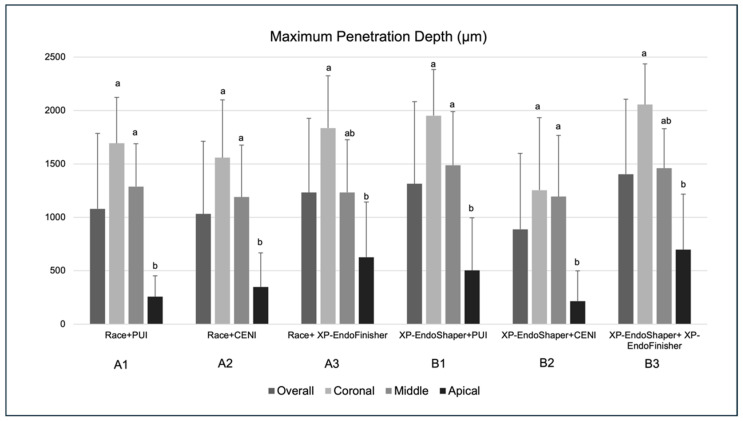
Maximum sealer penetration depth (µm) ± standard deviation measured by CLSM in the six experimental groups (*n* = 10 per group) overall and at coronal, middle, and apical root levels. The Kruskal–Wallis test revealed no statistically significant differences among overall groups and among groups within the same root region (*p* > 0.05). Within-group analysis showed significantly lower penetration percentages in the apical region compared with coronal and/or middle regions (groups sharing a lowercase letter are not significantly different; *p* > 0.05).

Group B3 showed the overall highest value of the maximum penetration depth (1404.34 ± 701.23 µm), while group B2 showed the lowest value (888.17 ± 710.18 µm) (Figure 4). No significant differences were detected in the overall maximum penetration depth among groups, as well as when considering the corresponding root regions among groups. As for the analysis within groups, maximum penetration depth was significantly lower in the apical region compared to the coronal region in all groups (*p* < 0.05). Additionally, values of the apical region were also significantly lower compared to the middle region in groups A1, A2, B1, and B2 (*p* < 0.05).

As for the multivariate analysis, the type of irrigant activation showed a significant effect on the maximum sealer penetration depth. Specifically, XP-EndoFinisher yielded the highest values, followed by PUI and CENI. The difference between the XP-EndoFinisher and CENI-activated groups was statistically significant (*p* = 0.02). On the other hand, the shaping method had no significant effect on the overall mean sealer penetration depth (*p* = 0.43).

### 3.3. Mean Percentage of Sealer Penetration

**Figure 5:**
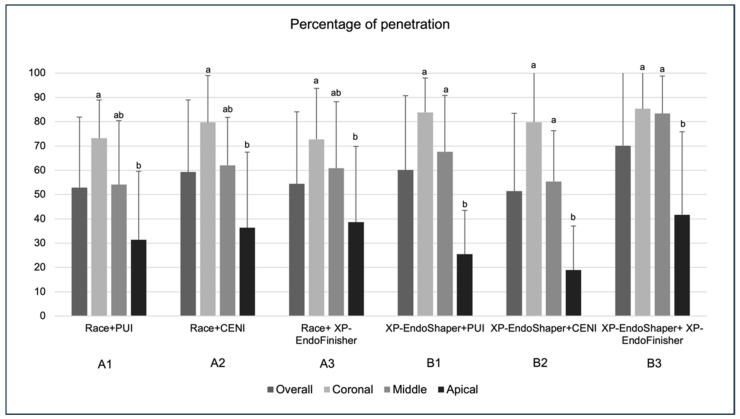
Percentage of sealer penetration into dentinal tubules (%) ± standard deviation in the six experimental groups (*n* = 10 per group) overall and at coronal, middle, and apical root levels. The Kruskal–Wallis test revealed no statistically significant differences among overall groups and among groups within the same root region (*p* > 0.05). Within-group analysis showed significantly lower penetration percentages in the apical region compared with coronal and/or middle regions (groups sharing a lowercase letter are not significantly different; *p* > 0.05).

With regard to the overall mean percentage of sealer penetration (Figure 5), the highest and lowest values were observed, respectively, in group B3 (70.16 ± 31.06%) and B2 (51.39 ± 32.03%). No significant differences were found among groups. As for the evaluation by root regions, no significant differences were observed in the inter-group comparison. Considering the analysis by root regions within groups, significantly lower values were found in the apical region compared to the coronal region in all groups (*p* < 0.05). In groups B1, B2, and B3, the values of apical sections were also significantly lower (*p* < 0.05) compared to the corresponding middle regions.

With regard to the multivariate analysis, neither the shaping nor the irrigant activation methods had a significant effect on the overall percentage of sealer penetration (*p* = 0.32 and *p* = 0.22, respectively).

### 3.4. Percentage of Sealer Integrity

**Figure 6:**
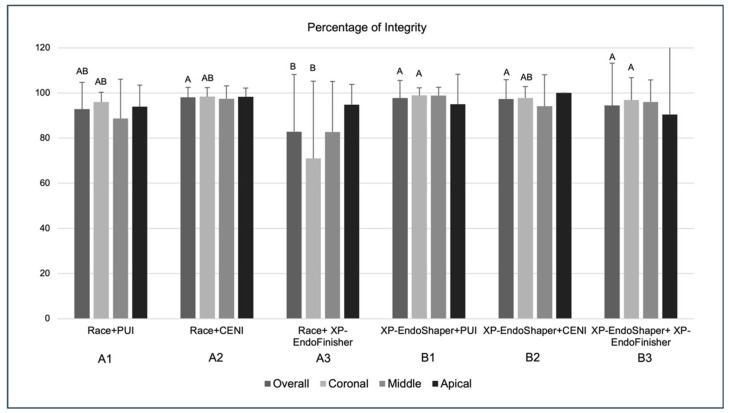
Percentage of sealer integrity on the canal walls (%) ± standard deviation in the six experimental groups (*n* = 10 per group) overall and at coronal, middle, and apical root levels. Inter-group comparisons showed statistically significant differences for overall sealer integrity among groups (Kruskal–Wallis test, *p* < 0.05), and pairwise comparisons were subsequently assessed using Dunn’s post hoc test with Bonferroni correction (groups sharing an uppercase letter are not significantly different; *p* > 0.05). Intra-group comparisons among root regions showed no statistically significant differences (*p* > 0.05).

The highest and lowest values for the percentage of sealer integrity on the canal walls were observed for group A2 (98.02 ± 4.5%) and group A3 (82.85 ± 25.35%), respectively (Figure 6). Considering all root regions, the overall percentage of sealer integrity on the canal walls was found to be significantly lower in group A3 as compared to groups A2 (*p* = 0.017), B1 (*p* = 0.004), B2 (*p* = 0.004), and B3 (*p* = 0.006). When considering the corresponding root canal regions among groups, significantly lower values were found in coronal sections of group A3 as compared to coronal sections from groups B1 (*p* = 0.026) and B3 (*p* = 0.034), while no significant differences were detected for other root regions. The comparison of sealer integrity in different root regions yielded no significant differences within groups.

As for the multivariate analysis, a significant interaction was found between shaping and activation methods (*p* = 0.05) on the percentage of sealer integrity. Specifically, the XP-Endo Shaper showed significantly higher values than Race (*p* = 0.003). Additionally, the CENI activation method yielded the highest values, followed by the PUI method, and lastly, the XP-Endo Finisher, with a statistically significant difference between CENI and the XP-Endo Finisher (*p* = 0.02).

**Figure 7:**
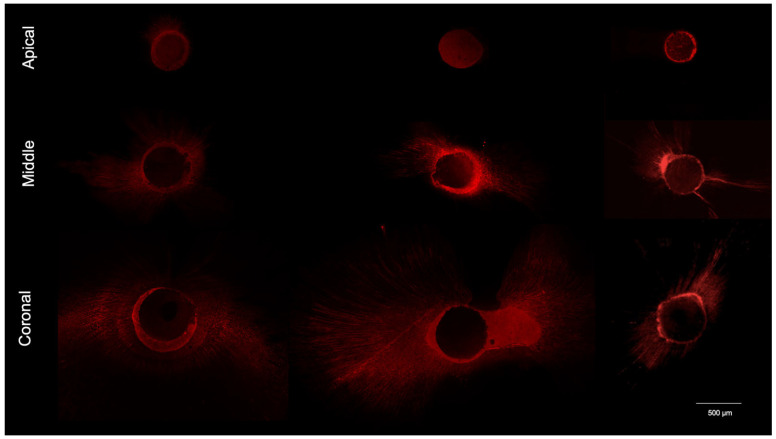
Representative images of confocal microscopy acquisitions (10× magnification) at apical, middle, and coronal levels. Image scale is defined by calibrated scale bars rather than nominal magnification to provide a more accurate and absolute spatial reference.

Representative images of confocal laser microscopy acquisitions are displayed in Figure 7. Raw data are reported in Supplementary Table S1 (mean values and standard deviations) and Table S2 (medians and interquartile range Q1–Q3).

## 4. Discussion

The stability and tridimensionality of root canal obturation are critical factors in the long-term clinical success of endodontic therapy, particularly in relation to the ability of obturation materials to penetrate dentinal tubules [24]. In the present in vitro study, we investigated the combined influence of shaping systems and irrigant activation methods on the intratubular penetration and peripheral integrity of a bioceramic sealer using CLSM analysis.

The null hypothesis was rejected, since significant differences were observed in specific outcome variables, while others did not differ significantly among groups. Overall, all the chemo-mechanical protocols tested yielded comparable results in terms of maximum penetration depth and percentage of sealer penetration, whereas differences emerged primarily for mean penetration depth and sealer integrity.

The combination of the XP-Endo Shaper and XP-Endo Finisher showed a tendency toward improved tridimensional sealer distribution, particularly in terms of mean penetration depth, although differences were not consistently significant across all evaluated parameters.

These results were also confirmed to some extent by the multivariate analysis, which identified a significant contribution of the type of irrigant activation to the sealer penetration depths, with significantly better results for the groups activated with the XP-Endo Finisher.

Similarly, regarding the mean percentage of sealer penetration, although no significant interactions were observed, the group treated with the combination of the XP-Endo Shaper and XP-Endo Finisher showed considerably higher values in the middle sections compared to the other groups, suggesting an improved tendency to obtain good sealer tridimensionality towards the deepest regions of the root canal.

Overall, these findings are likely due to the synergy between the XP-Endo Shaper’s adaptive technology and the XP-Endo Finisher’s specialized morphology and kinematics, which together enhance the canal’s chemo-mechanical preparation. From a mechanistic standpoint, the observed trends may be explained by differences in irrigant hydrodynamics and canal wall contact rather than by shaping geometry alone. Mechanical activation with the XP-Endo Finisher likely increases shear stress and irrigant renewal at the dentin–sealer interface, facilitating smear layer disruption and enhancing sealer flow into patent tubules. Conversely, conventional needle irrigation is limited by stagnant zones and reduced apical fluid exchange, which may explain its consistently lower penetration values across parameters.

Clinically, this suggests that adaptive shaping systems used in association with mechanical irrigant activation could be especially beneficial in cases where enhanced disinfection and sealing of the middle and apical thirds are critical, such as necrotic pulps, retreatments, or anatomically complex canals [10].

Comparing our findings to the existing literature, while intratubular sealer penetration has been previously studied in the context of specific variables such as irrigation protocols, shaping techniques, and obturation methods, few studies have explored the combined effects of different shaping protocols and irrigation techniques [10,25,26]. Although many studies found no significant differences in intratubular penetration values among various irrigant activation techniques, some authors found a greater depth of penetration with active irrigant activation, compared to conventional activation [10,25,27,28]. Our findings generally align with such literature, as well as with a common higher mean penetration, maximum penetration, and penetration percentages in coronal sections compared to apical ones. The consistently lower sealer penetration observed in the apical third across all protocols highlights the persistent clinical challenge of apical disinfection and sealing. The presence of a decreasing gradient in the coronal–apical direction seems to be independent of the type of shaping performed, the type of endodontic sealer, the obturation technique used, the presence/absence of the smear layer, the type of irrigants, and their eventual activation [12,13,24,29]. The decrease in the diameter and density of dentinal tubules at the apical level that leads to a reduction in the permeability and sealer flow might explain these findings. Additionally, dentin is more mineralized and sclerotic in the apical third, and the smear layer may be more challenging to remove, thereby hindering sealer and irrigant penetration [25,30]. In contrast, higher penetration in coronal sections may result from the greater compressive force near the canal orifice exerted during obturation, which produces a stronger lateral force leading the sealer into tubules [30]. Furthermore, the efficacy of smear layer removal is typically higher in coronal sections, as irrigants are less effective in the apical regions due to limited reach and fluid dynamics constraints [4].

Regarding the percentage of sealer integrity, despite improved intratubular penetration, the reduced sealer integrity observed with the RACE + XP-Endo Finisher combination suggests that not all activation techniques are universally beneficial, and that compatibility between shaping systems and activation devices should be considered when planning a chemo-mechanical protocol. These findings also suggest that the use of the XP-Endo Finisher System should be complemented with the corresponding XP-Endo Shaper to achieve good performance in terms of sealer integrity, possibly due to the unique design of these instruments. Interestingly, however, the percentage of sealer integrity around the perimeter of the canal was similar at each root level, demonstrating the maintenance of seal quality throughout the canal, which is critical to ensure a good clinical outcome. Additionally, though beyond the scope of this study, the generally high percentage of sealer integrity across all root levels supports the clinical reliability of bioceramic sealers used with a single-cone technique, provided that adequate chemo-mechanical preparation and irrigant activation are performed.

Overall, these results may have clinical relevance and indicate that active irrigant activation strategies may offer incremental benefits without increasing procedural complexity, potentially improving long-term treatment outcomes while maintaining conservative canal preparation.

The discrepancies noted between this study and others may be due to the use of different instrumentation systems, the type and concentration of irrigants used, the possible use of calcium chelators, the type of sealer used and the obturation technique, the choice of the section location, especially for apical sections where 1 mm can make a significant difference, or, finally, the sensitivity of the operator [10,12,31,32,33,34,35]. Future research should validate the clinical relevance of these findings and test the impact of different canal curvatures, sealer formulations, and obturation pressures.

**Figure 8:**
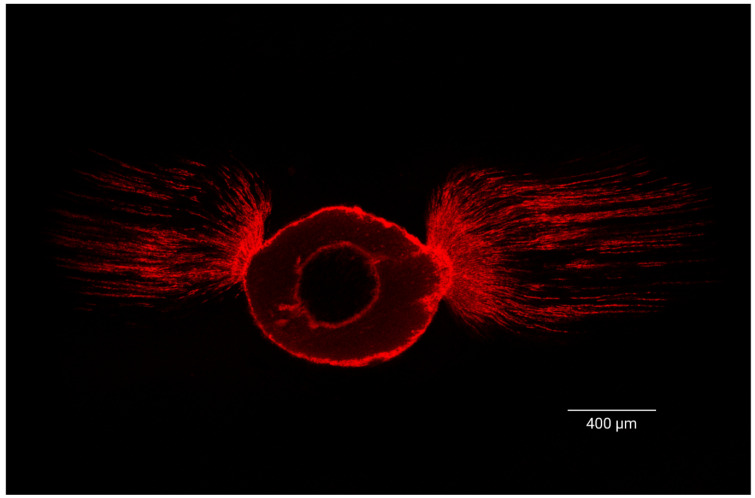
Representative sample displaying the “butterfly effect”. Image scale is defined by calibrated scale bars rather than nominal magnification to provide a more accurate and absolute spatial reference.

An additional consideration is the “butterfly effect,” a phenomenon in which dentinal tubules exhibit an asymmetric distribution along the buccal–lingual versus mesio-distal directions, observed in some of our sections, at each of the levels investigated (Figure 8). This pattern, frequently seen in mono-radicular teeth, resembles a butterfly shape in cross-sectional views, with greater tubule density along the buccal–lingual plane and a lower density of pervious dentinal tubules along the mesio-distal surfaces of the canal due to dentinal sclerosis, as demonstrated by Russell’s studies [36,37]. As a result, sealer penetration will presumably be greater in the buccal–lingual direction than in the mesio-distal direction, which can significantly impact penetration measurements.

In this regard, the present study has a number of limitations. First, this study lacks donor age information. Age-related dentinal sclerosis is known to reduce dentinal patency and permeability and may influence the apparent extent of sealer penetration [38]. Although the random allocation of specimens likely distributed this variable evenly among groups, absolute penetration values should be interpreted with caution. Future studies could benefit from a controlled sample population, ideally of a similar age range, to minimize the confounding effects of sclerosis on sealer penetration. In addition, the exclusion of samples displaying dentinal sclerosis or the “butterfly effect” by micro-CT or microscopic screening could reduce outcome variability, though this would require larger sample sizes to ensure statistical power. Alternatively, a refined approach to calculating the average penetration by excluding null mesio-distal measurements in such samples or incorporating age-stratified analyses could provide more accurate estimates without compromising the sample size. An additional limitation of this study is that Rhodamine B, although commonly used for its simplicity and absence of chemical interactions with sealers, may have influenced the sealer flow behavior to some extent [39,40]. Rhodamine B is known to diffuse in dentin, which may inflate absolute penetration metrics [41]. Accordingly, the present CLSM outcomes should be interpreted as apparent tracer penetration associated with sealer distribution rather than a direct quantification of true intratubular sealer penetration. Nevertheless, because all groups underwent identical staining and imaging protocols, the comparative differences observed among shaping procedures remain meaningful, while absolute values should be interpreted with caution. Furthermore, although measurements were performed using standardized protocols by the same blinded operator, intra-operator reliability was not formally tested, and the operator was not blinded to the clinical procedure, which may introduce operator-related bias. These aspects should be addressed in future investigations.

Finally, the in vitro design does not replicate the complex biological and biomechanical conditions present in vivo, such as the periodontal ligament and microbial biofilms, which may influence the sealer behavior [42]. These limitations should be carefully considered when extrapolating the present findings to clinical practice.

## 5. Conclusions

These findings highlight the importance of shaping and irrigation techniques in optimizing sealer penetration and the need to consider anatomical and technical variables in endodontic studies to better predict clinical outcomes. Within the limitations of this in vitro study, the findings suggest that irrigant activation, particularly mechanical activation with the XP-Endo Finisher, may enhance the tridimensional distribution of a bioceramic sealer, especially in the middle and apical thirds of the root canal. These results should be interpreted as indicative of potential trends rather than definitive evidence for clinical superiority. Further clinical and ex vivo studies are required to confirm the relevance of these observations under in vivo conditions.

## Figures and Tables

**Table 1:** Protocols of chemo-mechanical shaping for experimental groups (PUI: passive ultrasonic irrigation; CENI: conventional needle irrigation).

Working Length and Glide Path	Groups Based on Different Chemo-Mechanical Shaping Protocols	Shaping Protocols	Irrigant Activation Protocols
C-file #10 at WL 2 mL NaOCl 5.25% Endowave MGP 10/.02 * 2 mL NaOCL 5.25% Endowave MGP 15/.02 * 2 mL NaOCL 5.25% Endowave MGP 20/.02 * 2 mL NaOCL 5.25% * 800 rpm, 0.4 N/cm torque	(A1) Race + PUI; (A2) Race + CENI; (A3) Race + XP-Endo Finisher; (B1) XP-Endo Shaper + PUI; (B2) XP-Endo Shaper + CENI; (B3) XP-Endo Shaper + XP-Endo Finisher	(A) Race Race 20/.04 * 2 mL NaOCl 5.25% Race 25/.04 * 2 mL NaOCl 5.25% Race 30/.04 * * 600 rpm, 1.5 N/cm torque (B) XP-Endo Shaper Xp-Endo Shaper * 3/5× to reach WL, immersed in NaOCl 5.25% 2 mL NaOCl 5.25% Xp-Endo Shaper * 15× to WL, immersed in NaOCl 5.25% * 1000 rpm, 1 N/cm torque	(1) PUI (EndoUltra) 1 mL NaOCl 5.25% ultrasound-activated 30 s 1 mL NaOCl 5.25% ultrasound-activated 30 s 1 mL EDTA 17% ultrasound-activated 30 s 1 mL EDTA 17% ultrasound-activated 30 s 2 mL saline
			(2) CENI 1 mL NaOCl 5.25% with side-vented needle at WL-2 mm, 30 s 1 mL NaOCl 5.25% with side-vented needle at WL-2 mm, 30 s 1 mL EDTA 17% with side-vented needle at WL-2 mm, 30 s 1 mL EDTA 17% with side-vented needle at WL-2 mm, 30 s 2 mL saline
			(3) XP-Endo Finisher 2 mL NaOCl 5.25% activated with XP-Endo Finisher *, 60 s 2 mL EDTA 17% activated with XP-Endo Finisher *, 60 s 2 mL saline * 1000 rpm and 1 N/cm

The asterisks (*) indicate that further details on the operating setting for the instrument are reported at the end of the corresponding column.

## Data Availability

The original contributions presented in this study are included in the article/Supplementary Material. Further inquiries can be directed to the corresponding author.

## Supplementary Materials

The following supporting information can be downloaded at https://www.mdpi.com/article/10.3390/jcm15031132/s1. Table S1: Values of mean penetration depth (µm), maximum penetration depth (µm), penetration percentage (%) and percentage (%) of sealer integrity on canal walls and standard deviations measured on confocal scanning laser microscopy images, presented as means ± standard deviations. Table S2: Values of mean penetration depth (µm), maximum penetration depth (µm), penetration percentage (%) and percentage (%) of sealer integrity on canal walls and standard deviations measured on confocal scanning laser microscopy images, presented as median ± interquartile range [IQR] (Q3–Q1).

## Abbreviations

The following abbreviations are used in this manuscript:

PUI	Passive ultrasonic irrigation
CENI	Conventional endodontic needle irrigation
PRILE	Preferred reporting items for laboratory studies in endodontology
CEJ	Cement–enamel junction
CLSM	Confocal laser scanning microscope
