# Supplementary material for: Effect of Different Chemo-Mechanical Shaping Protocols on the Intratubular Penetration of a Bioceramic Sealer
Source: J Clin Med. 2026 Feb 1;15(3):1132. doi: 10.3390/jcm15031132 (PMC12898361; doi:10.3390/jcm15031132)
Supplement: Supplementary file 1 [file jcm-15-01132-s001.zip › jcm-4098180-supplementary.pdf]

Journal of Clinical Medicine

MDPI

Table S1. Values of mean penetration depth (μm), maximum penetration depth (μm), penetration percentage (%) and percentage (%) of sealer integrity on canal walls and standard deviations measured on confocal scanning laser microscopy images, presented as mean± standard deviation.

	A1 (*n*=10)	A2 (*n*=10)	A3 (*n*=10)	B1 (*n*=10)	B2 (*n*=10)	B3 (*n*=10)
	Race + PUI	Race + CENI	Race + XP-EndoFinisher	XP-EndoShaper + PUI	XP-EndoShaper + CENI	XP-EndoShaper + XP-EndoFinisher
	(mean ± SD)	(mean ± SD)	(mean ± SD)	(mean ± SD)	(mean ± SD)	(mean ± SD)
**Mean penetration depth (μm)**						
Coronal	520.56 ± 205.85	660.48 ± 364.62	820.05 ± 493.19	722.83 ± 352.44	504.4 ± 381.22	1005.94 ± 434.97
Middle	306.59 ± 227.95	406.84 ± 344.07	449.33 ± 381.58	558.07 ± 350.17	351.5 ± 251.11	672.7 ± 308.37
Apical	70.77 ± 86.6	110.79 ± 141.88	186.8 ± 217.2	136.49 ± 203.25	46.96 ± 85.28	234.89 ± 277.36
Overall	299.31 ± 257.9	392.7 ± 369.38	485.4 ± 452.91	472.46 ± 390.5	300.95 ± 323	637.84 ± 463.93
**Maximum penetration depth (μm)**						
Coronal	1694.1 ± 428.86	1558.32 ± 541.69	1835.98 ± 488.78	1951.49 ± 432.96	1254.43 ± 679.77	2055.89 ± 380.46
Middle	1288.48 ± 401.47	1190.41± 485.75	1233.83 ± 492.3	1488.46 ± 501.78	1194.15 ± 572.49	1460.21 ± 370.22
Apical	257.23 ± 196.29	348.1± 319.96	626.6 ± 516.31	503.72 ± 493.41	215.93 ± 283.06	696.93 ± 520.98
Overall	1079.94 ± 705.35	1032.28 ± 679.36	1232.14 ± 695.89	1314.56 ± 767.4	888.17 ± 710.18	1404.34 ± 701.23
**Penetration percentage (%)**						
Coronal	73.18 ± 15.8	79.76 ± 19.28	72.74 ± 20.96	83.82 ± 14.19	79.8 ± 21.33	85.36 ± 18.84
Middle	54.11 ± 26.3	62.06 ± 19.78	60.87 ± 27.35	67.64 ± 23.14	55.42 ± 20.92	83.4 ± 15.38
Apical	31.45 ± 28.13	36.37 ± 31.14	38.65 ± 31.22	25.47 ± 18.05	18.95 ± 18.13	41.71 ± 34.16
Overall	52.91 ± 28.96	59.29 ± 29.71	54.42 ± 29.62	60.13 ± 30.61	51.39 ± 32.03	70.16 ± 31.06
**Integrity percentage (%)**						
Coronal	95.95 ± 4.33	98.37 ± 40	71.02 ± 34.21	98.95 ± 3.32	97.79 ± 5	96.87 ± 9.88
Middle	88.64 ± 17.5	97.44 ± 5.73	82.7 ± 22.46	98.85 ± 3.65	94.09 ± 13.92	96.05 ± 9.67
Apical	93.94 ± 9.58	98.26 ± 3.96	94.83 ± 9.02	95.05 ± 13.23	100 ± 0	90.47 ± 30.13
Overall	92.84 ± 11.8	98.02 ± 4.5	82.85 ± 25.35	97.71 ± 7.82	97.29 ± 8.6	94.47 ± 18.69

Journal of Clinical Medicine MDPI

Table S2. Values of mean penetration depth (μm), maximum penetration depth (μm), penetration percentage (%) and percentage (%) of sealer integrity on canal walls and standard deviations measured on confocal scanning laser microscopy images, presented as median ± interquartile range [IQR] (Q3-Q1).

	A1 (*n*=10)	A2 (*n*=10)	A3 (*n*=10)	B1 (*n*=10)	B2 (*n*=10)	B3 (*n*=10)
	Race + PUI	Race + CENI	Race + XP-EndoFinisher	XP-EndoShaper + PUI	XP-EndoShaper + CENI	XP-EndoShaper + XP-EndoFinisher
	(median [IQR])	(median [IQR])	(median [IQR])	(median [IQR])	(median [IQR])	(median [IQR])
**Mean penetration depth (μm)**						
Coronal	449.75 [92.84]	632.60 [373.97]	705.25 [826.96]	627.45 [282.81]	298.51 [589.01]	1021.73 [627.64]
Middle	251.07 [297.205]	338.29 [374.92]	276.25 [649.38]	501.65 [391.15]	296.48 [428.55]	637.53 [436.07]
Apical	45.09 [88.57]	34.81 [242.68]	91.23 [372.44]	41.22 [107.11]	10.30 [63.21]	114.07 [460.99]
Overall	272.32 [358.07]	263.62 [503.41]	358.07 [603.48]	464.42 [521.45]	228.45 [340.10]	564.17 [739.52]
**Maximum penetration depth (μm)**						
Coronal	1725.62 [451.44]	1542.401 [979.84]	1868.89 [644.81]	1807.36 [601.81]	1178.72 [1269.47]	2071.43 [548.92]
Middle	1377.68 [780.294]	1222.53 [574.67]	1091.22 [474.70]	1533.78 [588.79]	1377.70 [1111.26]	1554.45 [508.93]
Apical	276.82 [432.02]	247.13 [690.65]	465.51 [549.41]	262.60 [535.81]	133.50 [237.74]	646.90 [909.83]
Overall	1143.65 [1219.46]	957.15 [993.57]	1222.79 [1074.88]	1490.79 [1244.21]	764.44 [1327.05]	1514.19 [833.51]
**Penetration percentage (%)**						
Coronal	74.34 [16.33]	79.73 [36.60]	74.08 [22.71]	85.35 [26.94]	85.60 [34.31]	91.33 [23.35]
Middle	57.48 [41.846]	63.43 [19.44]	60.13 [46.69]	63.01 [43.52]	60.99 [40.54]	86.19 [34.25]
Apical	27.03 [44.02]	30.31 [47.82]	33.91 [52.38]	24.88 [24.04]	15.31 [25.04]	37.17 [65.43]
Overall	60.07 [45.40]	63.40 [38.06]	62.15 [45.10]	60.54 [51.63]	55.56 [52.29]	81.37 [54.50]
**Integrity percentage (%)**						
Coronal	96.37 [6.50]	100 [0]	90.47 [56.24]	100 [0]	100 [0]	100 [0]
Middle	100 [27.99]	100 [0]	95.68 [36.25]	100 [0]	100 [0]	100 [0]
Apical	100 [10.54]	100 [0]	100 [12.51]	100 [0]	100 [0]	100 [0]
Overall	100 [9.68]	100 [0]	95.82 [25.71]	100 [0]	100 [0]	100 [0]
